# EHMTI-0162. Cognitive behavioural treatment for the chronic post-traumatic headache patient: a randomised controlled trial

**DOI:** 10.1186/1129-2377-15-S1-C29

**Published:** 2014-09-18

**Authors:** D Kjeldgaard Nielsen, H Forchhammer, TW Teasdale, RH Jensen

**Affiliations:** 1 Department of Neurology, Danish Headache Center University of Copenhagen, Glostrup, Denmark; 2 Department of Neurology, University of Copenhagen, Glostrup, Denmark; 3 The Department of Psychology, Faculty of Social Sciences University of Copenhagen, Copenhagen, Denmark

## Background

Chronic post-traumatic headache (CPTH) after mild head injury can be difficult to manage. Research is scarce and successful interventions are lacking.

## Aim

To conduct a randomized controlled trial (RCT) exploring whether a group-based Cognitive Behavioural Therapy (CBT) intervention would lead to a relative decrease in headache, pain perception and psychological symptoms and an increase in quality of life in the study group compared to a waiting-list control group.

## Methods

Ninety patients with CPTH according to ICHD-2 criteria were enrolled from the Danish Headache Center. Patients were randomly assigned to either a waiting- list group or to a nine-week CBT group intervention. At baseline and after 26 weeks all patients completed the Rivermead Post-concussion Questionnaire, SF-36, SCL-90-R (psychological distress) and a headache diary.

## Results

The CBT had no effect on headache and only a minor impact on the CPTH patients' quality of life, psychological distress, and the overall experience of symptoms. The waiting-list group had also unchanged headache but, opposed to the treatment group, a significant decrease in somatic and cognitive symptoms indicating a spontaneous remission over time.

## Conclusions

Our primarily negative findings confirm that management of patients with CPTH still remains a considerable challenge. Psychological group therapy with CBT might be effective in an earlier stage of CPTH and in less severely affected patients. There is an urgent need for development of new treatment strategies and a need for randomized controlled studies to test the efficacy of psychological therapy for CPTH patients.

No conflict of interest.

